# Oral Health Literacy In Young Migrant/Refugee Mothers From Developing Countries

**DOI:** 10.1093/eurpub/ckac129.259

**Published:** 2022-10-25

**Authors:** J Esimekara, H Lee

**Affiliations:** 1 Unité de Chirurgie Orale, Département de Chirurgie Hôpitaux Universitai, Geneva, Switzerland; 2 Oral Health Workgroup, WFPHA, Geneva, Switzerland

## Abstract

**Background and problems:**

In the last decade, migration has increased due to climatic, economic, and social factors. Oral health is an integral part of overall health, but primary health packets for migrants/refugees often neglect oral health education and care in the care delivery system. The present article aims to present a realistic approach to integrating oral health into existing structures for young migrant/refugee mothers on a community level in Switzerland.

**Importance:**

As a young child's oral health is closely related to mother's oral health status and oral health knowledge level, it would be critical that migrant/refugee mothers have access to timely oral health education and care through the primary health care system.

**Solution:**

We propose an integrated oral health care model for migrant/refugee mothers that maximizes the existing primary health care system. Using existing maternal oral health resources, a training module is developed for the primary care care workforce that serve migrant/refugee mothers. This training module includes oral health needs and status assessment and delivering preventive oral health services in primary care settings. The module includes drawings and images to describe the dangers of poor oral hygiene during pregnancy related to the health of mothers and babies and how to keep the mouth healthy with low-cariogenic diet practice and home oral hygiene care. Primary health care workforce in selected migrant health centers, like nurses, medical aides, and community health care workers, is trained by this module and evaluated by a set of self-assessed questionnaires before and after the training for knowledge improvement, attainment, and clinical practice changes. Migrant mothers participate in questionnaires on oral health hygiene practice and access to dental care during pregnancy and for their young children. The questionnaires are validated by the affiliated university-hospital clinical research center.

